# Functional analysis and evaluation of respiratory cilia in healthy Chinese children

**DOI:** 10.1186/s12931-020-01506-w

**Published:** 2020-10-09

**Authors:** So-Lun Lee, Christopher O’Callaghan, Yu-Lung Lau, Chun-Wai Davy Lee

**Affiliations:** 1 Department of Paediatrics and Adolescent Medicine, Queen Mary Hospital, HKSAR, China; 2 Department of Paediatrics and Adolescent Medicine, Duchess of Kent Children’s Hospital, HKSAR, China; 3 Respiratory, Critical Care and Anaesthesia, UCL Great Ormond Street Institute of Child Health and GOSH NIHR BRC, London, UK; 4 Department of Paediatrics and Adolescent Medicine, The University of Hong Kong, HKSAR, China

**Keywords:** Beat frequency, Beat pattern, Chinese children, Nasal cilia, Reference data, Ultrastructure

## Abstract

**Background:**

To aid in the diagnosis of Primary Ciliary Dyskinesia (PCD) and to evaluate the respiratory epithelium in respiratory disease, normal age-related reference ranges are needed for ciliary beat frequency (CBF), beat pattern and ultrastructure. Our aim was to establish reference ranges for healthy Chinese children.

**Methods:**

Ciliated epithelial samples were obtained from 135 healthy Chinese children aged below 18 years by brushing the inferior nasal turbinate. CBF and beat pattern were analysed from high speed video recordings. Epithelial integrity and ciliary ultrastructure were assessed using transmission electronic microscopy.

**Results:**

The mean CBF from 135 children studied was 10.1 Hz (95% CI 9.8 to 10.4). Approximately 20% (ranged 18.0–24.2%) of ciliated epithelial edges were found to have areas of dyskinetically beating cilia. Normal beat pattern was observed in ciliated epithelium from all subjects. We did not find any effect of exposure to second hand smoke on CBF in our subjects. Microtubular defects were found in 9.3% of all of the cilia counted in these children, while other ciliary ultrastructural defects were found in less than 3%.

**Conclusions:**

We established the reference range for CBF, beat pattern and ultrastructure in healthy Chinese children. Using similar methodology, we found a lower overall mean CBF than previously obtained European values. This study highlights the need to establish normative data for ciliary function in different populations.

## Introduction

Primary ciliary dyskinesia (PCD) is a rare genetic condition with ciliary ultrastructural defects leading to ineffective ciliary movement and impaired mucociliary clearance. Patients with PCD develop recurrent upper and lower chest infections leading to bronchiectasis, impaired lung function and respiratory failure [1, 2]. Many patients have persistent symptoms from birth or early infancy but the diagnosis is often made at a late stage [3]. Early diagnosis with appropriate management may reduce morbidity, preserve lung structure and lung function.

However, there is no gold standard for the diagnosis of PCD and there are significant disparities between international guidelines. European Respiratory Society (ERS) PCD diagnostic task force recommends a combination of nasal nitric oxide (nNO) measurement and digital high speed video microscopy (HSVM) assessment of ciliary beat frequency (CBF) and pattern as the first step in diagnosing PCD [4]. American Thoracic Society (ATS) puts more emphasis on the use of nNO and an extended genetic panel and suggests not to use HSVM [5]. Both guidelines agreed that definitive diagnosis should be made with hallmark ciliary ultrastructural defect on transmission electron microscopy (TEM) or biallelic pathogenic variants in PCD associated genes. To reduce confusion, the ERS PCD diagnostic task force and ATS worked together to identify the reasons for the discrepancies and highlighted the steps need for them to improve agreement on diagnostic criteria. They also suggested that research to develop an internationally agreed diagnostic pathway for PCD was needed, along with standardization of operational procedures and quality control of diagnostic tests being one of the major recommendations [6].

CBF can be measured by photomultiplier and photodiode techniques but the values are significantly lower than by HSVM [7]. HSVM has the additional advantage of allowing analysis of beat pattern that has been shown to be abnormal in PCD [8]. HSVM has been shown to have a high sensitivity and specificity for the diagnosis of PCD [9–11]. However, secondary epithelial damage following a viral infection [12] and in severe asthma may result in significant secondary ciliary dyskinesia [13, 14].

TEM is regarded as a highly specific test to confirm PCD. Nevertheless, up to 30% of PCD cases may have an apparently normal or non-diagnostic ultrastructure [6, 15]. Moreover, causative genetic mutations are seldom identified with variance of unknown significance commonly found in PCD genes. There is no doubt that the inclusion of all four diagnostic tests will increase the diagnostic accuracy of PCD.

The collection, processing and interpretation of ciliary biopsy samples for both HSVM and TEM require special expertise and are preferably conducted in specialized centres. Normal ranges are essential to help interpret the findings and should be established for individual centres as it has been shown that baseline CBF values could be different across centres in the United Kingdom (UK) despite the adoption of similar protocol [16]. In addition, normal data on ciliary beat pattern has only been reported for a European population [1]. Our aim was to establish normal age-related reference ranges for CBF and pattern using digital HSVM and ultrastructure of the ciliated epithelium and ciliary axonemes using TEM in Chinese children. It also allowed for comparison with normative data from a European study using the same methodology.

## Methods

### Study design and subjects

For this cross-sectional study, healthy Chinese school children aged less than 18 years and adult volunteers in Hong Kong (HK) were recruited. Subjects with a history of chronic respiratory or nasal disease, symptomatic upper respiratory tract infection (URTI) in the previous 6 weeks, those requiring long-term medication or known cigarette smokers were excluded [1]. Exposure to second hand smoking (SHS) at home was explored. Subjects were examined to exclude obvious nasal defects with an auroscope. The inferior turbinate of subjects was brushed with a 3 mm cytology brush (Conmed, product no. 149R) to obtain nasal epithelial cells, without topical anaesthetics. During the procedure, subjects were shown a video on a mobile phone as distraction. Nasal brushings were placed in medium 199 (pH 7.3) supplemented with streptomycin 50 μg/ml and penicillin 50 IU/ml (Gibco, UK).

### Analysis of CBF and beat pattern by HSVM [1, 8] and ultrastructure by TEM [9]

Ciliated strips of epithelium were suspended in a chamber using a glass slide and cover slips. The slide was placed on a heated stage (37 °C) of a Leica light microscope mounted on an anti-vibration table. Specimens were examined using a × 100 interference contrast lens. Undisrupted ciliated strips of about 50 μm in length and devoid of mucus were studied. Beating ciliated edges from side-view profile (10 video chips), above-view profile (2 video chip) and towards the observer profile (1 video-chip), were recorded using a digital high speed video camera (IDT, model NX4) at a rate of 500 frames per second. The recorded side-view video sequences were projected onto a high resolution monitor and were played back at reduced frame rates or frame by frame to determine CBF. The ciliated edge was divided into 5 adjacent areas measuring 10 μm. Two measurements of CBF were recorded in each area, resulting in a total of 10 measurements for each edge. A maximum of 10 edges were analysed per subject. The number of frames for groups of beating cilia required to complete 10 cycles were recorded. CBF was converted using a calculation (CBF = 500/(number frames for 10 beats) × 10). The reproducibility for inter-observer (measured independently by two observers) and intra-observer (measured by the same observer after 2 days) CBF measurements were evaluated [1]. Normal ciliary beat pattern denoted a coordinated cilium beating in a forward and backward motion along the whole epithelial edge on side-view profile. Edges with dyskinetically beating cilia were counted and the percentage of these edges was calculated. Abnormal patterns, such as circular beating identified on above-view profile and towards the observer profile, were reported qualitatively.

### Transmission electron microscopy analysis

Nasal brushing samples were fixed in 2% glutaraldehyde and processed through resin using standard techniques [1]. Ultrathin sections were cut at 100 nm, collected on 200 mesh thin bar copper grids, stained in 1% uranyl acetate and counterstained in Reynold’s lead phosphate.

### Evaluation of ciliary structure and function

The ultrastructural changes of the epithelium and cilia were assessed in a blinded fashion [7]. The distal and proximal regions of the axoneme were examined. Dynein arms were assessed in cross sections with clear structural features and an intact ciliary membrane. The number of ciliated cells, mucous cells, and dead cells of the epithelium were expressed as a percentage of the total number of cells examined. Disruption and damage to the tissue was quantified using a scoring system that assessed the degree of loss of cilia from ciliated cells, projection of cells from epithelial edge, cytoplasmic blebbing and mitochondrial damage with a summation epithelial integrity that incorporated all of these factors to assess the overall epithelial damage: 0 = no damage, 1 = minor, 2 = mild, 3 = moderate, 4 = major, 5 = severe damage [1]. Cilia in less than perfect cross-section but in which the microtubular arrangement could be recognised were recorded as being normal (9 + 2 microtubules) or defective. The results were recorded in batches of 100 counts, assembling as many such batches from a single section, and thus any pattern would be identified. Dynein arms were assessed in any high-quality cross-sections encountered during the recording of microtubular arrangements and documented as showing the presence of both arms, only an outer or inner arm, or neither arm. If the presence of an arm was equivocal, it was counted as being present [17].

### Data analysis

In calculating the sample size, in order to establish age-related references in children aged < 18 years with a local population size of around 1,000,000 [18], allowing 9% margin of error from the mean at 5% confidence level, 120 subjects were required. This sample size of 120 subjects would have a power of 80 and 5% level of significance (two-sided) to detect a 10% difference of mean CBF between the Chinese and European population [1]. Assuming 10% of sample insufficiency and loss during preparation, a total of 135 subjects were recruited. The mean and standard deviation (SD), 5th and 95th percentiles of CBF, the mean percentage, 5th and 95th percentiles of edges exhibiting dyskinetically beating cilia, cells with loss of cilia, cellular projections, cytoplasmic blebbing, mitochondrial damage and ultrastructural defects including microtubular, dynein arm or other defects of the 3 individual age groups and the whole group were calculated. A one way analysis of variance (ANOVA) was performed to detect a significant difference among individual groups. The mean CBF between children < 18 years and adults with and without SHS exposure in each individual age group and between subjects in the current study and the European study were compared using t-test. Inter-observer and intra-observer difference of CBF measurement were calculated by comparing the 95% confidence interval (CI).

## Results

### Analysis of ciliary beat frequency and beat pattern measurements

We recruited 164 children (88 males, age range 2–17 years) and 50 adult volunteers from December 2015 to November 2016. We excluded 35 subjects because of known underlying diseases (11 children with allergic rhinitis [AR] with nasal congestion), URTI in previous 6 weeks (4 children with URTI alone and 11 also had underlying AR; 3 adults with URTI in previous 6 weeks alone and 2 also had underlying AR), inadequate samples (3 children) and being an active smoker (1 adult). Three of the 24 subjects with AR excluded were on intranasal medication. Samples from the remaining 135 children (67 males, age range 3–17 years) and 44 adults (25 males, age range 18–60 years) were analysed. The samples were processed for HSVM analysis and fixed for subsequent TEM with a mean of 5.5 h (range 1.5–11.5 h) from the time of nasal brushings [19]. Samples of 11 children aged 2–6 years and 10 children aged 13–17 years were processed at 9 to 11.5 h. Forty six children were exposed to SHS at home. Ten adults were exposed to SHS at home while 34 were not. The mean CBF and the percentage of dyskinetically beating edges for all subjects were summarized in Table 1. No significant difference was found in mean CBF (ANOVA, *p* = 0.542) and dyskinetically beating edges between the individual age groups (ANOVA, *p* = 0.212). The normal ciliary beat pattern from a subject showed coordinated cilia beating in a forward and backward motion along the whole epithelial edge (see Video 1) and the ciliated edges analysed that exhibited areas of dyskinetically beating cilia ranged from 18.0 to 24.2% (representative images of ciliated edge exhibited static cilia, see Video 2). There was also no significant difference in mean CBF between children with and without exposure to SHS for the 3 different age groups (ANOVA, *p* = 0.89 for children aged 2–6, *p* = 0.29 for children aged 7–12, *p* = 0.58 for children aged 13–17) but there was slightly higher mean CBF in adults with SHS exposure compared to adults without (ANOVA, *p* = 0.04) (Table 2). The mean CBF for children aged < 18 years was slightly higher than the adult group but it did not reach statistical significance [10.1 Hz (95% CI 9.8 to 10.4) versus 9.5 Hz (95% CI 8.9 to 10.0), ANOVA, *p* = 0.05]. Yet, it was lower than that of the European study [12.8 Hz (95% CI 12.3 to 13.3, ANOVA, *p* < 0.05)] [1]. There was no significant difference in mean CBF for samples processed within the plateau range of 3 to 9 h from collection [19] and those not within the plateau range for children aged < 18 years [10.52 Hz (95% CI 10.09 to 10.96) versus 10.50 Hz (95% CI 9.78 to 11.21), *p* = 0.969] and for adults [10.49 Hz (95% CI 9.81 to 11.16) versus 9.34 Hz (95% CI 8.44 to 10.24), *p* = 0.06].

**Table 1:** Analysis of ciliary beat frequency and beat pattern measurements

Age (years)	N	Mean^a^	SD	5th, 95th percentiles	Dyskinetically beating edges (%)^b^
< 18	135	10.1	2.0	6.3, 13.5	20.9 (0.0, 56.4)
2–6	51	10.3	2.0	6.9, 13.8	24.2 (0.0, 60.0)
7–12	43	10.1	2.1	5.8, 13.5	18.0 (0.0, 50.0)
13–17	41	9.9	1.9	6.1, 13.1	19.8 (0.0, 50.0)
> 18	44	9.5	1.9	5.4, 11.9	14.9 (0.4, 44.7)

^a^Mean ciliary beat frequency (Hz), standard deviation (SD), and 5th, 95th percentiles

^b^mean (5th, 95th percentiles) percentage of edges exhibiting areas of ciliary dyskinesia

UK reference CBF mean **f**or < 18 years 12.8 (95% CI 12.3 to 13.3)^1^ vs ours < 18 mean CBF 10.1 (95% CI 9.8 to 10.4) (*p* < 0.05, t-test).

**Table 2:** Effect of exposure to second-hand smoking on ciliary beat frequency

Age (years)	Family member(s) smoked		No		
	Yes				
	N	*Mean + SD	N	*Mean + SD	*P* value
< 18	46	9.9 + 1.68	89	10.2 + 2.16	0.284
2–6	11	10.4 + 1.53	40	10.3 + 2.09	0.886
7–12	21	9.7 + 2.13	22	10.4 + 2.15	0.289
13–17	14	9.7 + 0.81	27	10.0 + 2.33	0.575
> 18	10	10.5 + 1.65	34	9.2 + 1.82	0.044


**Additional file 1.** Video 1 High speed video images of ciliary function from a subject showing normal beating pattern


**Additional file 2**: Video 2 High speed video images of ciliary function from a subject showing ciliated edge exhibited static cilia

For ciliary beat pattern, 1 subject in the 13–17 year group had a mixed ciliary beat pattern with dyskinetic cilia, cilia with a normal pattern and cilia with a circular beat pattern when viewed from above on occasional ciliated cells (see Video 3). The mean CBF and TEM were normal. Normal beat pattern was observed in the ciliated epithelium from all other subjects (representative images of normal ciliary beat pattern from above and towards the observer view, see Video 4 and Video 5).


**Additional file 3** Video 3 High speed video images of ciliary function from a subject showing a mixed ciliary beat pattern with dyskinetic cilia, cilia with a normal pattern but with a circular beat pattern when viewed from above on occasional ciliated cells


**Additional file 4** Video 4 High speed video images of ciliary function from a subject showing normal beating pattern from above-view


**Additional file 5** Video 5 High speed video images of ciliary function from a subject showing normal beating pattern from towards the observer view

**Fig. 1:**
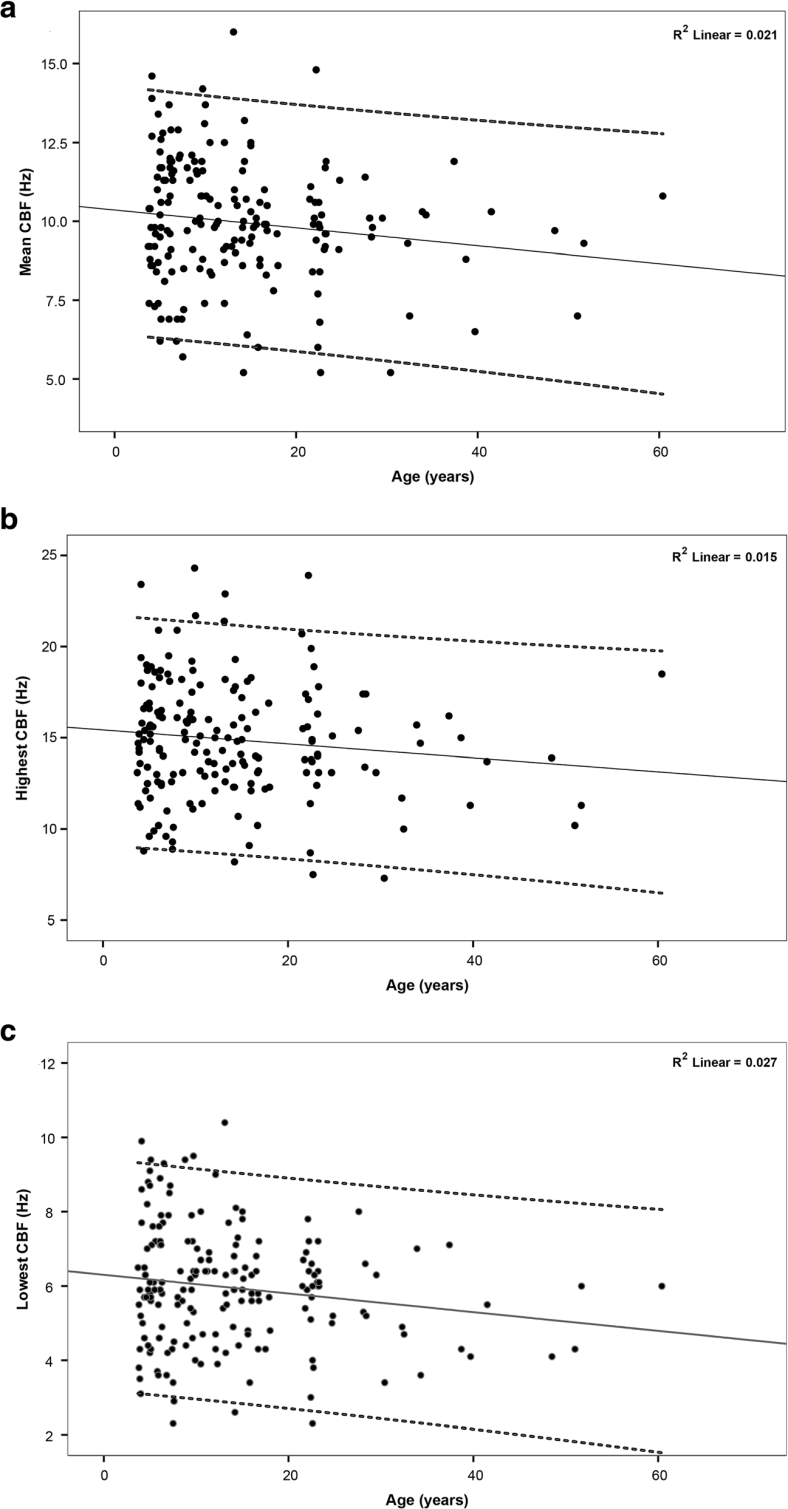
Relationship of the ciliary beat frequency (CBF) with age (years). **a** Mean CBF plotted against age for all subjects showing a negative correlation between increasing age and a reduction in CBF. Mean (solid line) and ± 1.96 standard deviation (dashed line) regression lines were indicated. **b** Ciliated edges with the highest and CBF within a nasal sample plotted against age for all subjects. Mean (solid line) and ± 1.96 standard deviation (dashed line) regression lines were indicated. **c** Ciliated edges with the lowest CBF within a nasal sample plotted against age for all subjects. Mean (solid line) and ± 1.96 standard deviation (dashed line) regression lines were indicated

To establish a reference range, mean CBF was plotted against the age of each subject (Fig. 1a). A weak negative correlation was found between mean CBF and increasing age (r^2^ = 0.021). As cilia were found to beat at different frequencies within each sample, we also plotted sample variation in CBF, the ciliated edges with the highest and lowest CBF against age. The highest mean CBF of edges ranged from 7.3 to 24.3 Hz (Fig. 1b) with 93% of subjects having a maximal CBF of > 10 Hz. The lowest mean CBF of edges ranged from 2.3 to 10.4 Hz (Fig. 1c) with 45% of subjects having a minimum CBF of > 6 Hz.

No significant difference was found for inter-observer and intra-observer measurements of CBF. The mean (SD) for inter-observer measurement was 0.57 (1.87) (95% CI − 0.77 to 1.9; range − 2 to 4) and intra-observer was 0.01 (1.4) (95% CI − 1.02 to 1.03; range − 3 to 3).

### Transmission electron microscopy examination of cell types

Some subjects had an inadequate sample for ultrastructural analysis as the tissue might have been lost during initial HSVM assessment and before the sample was processed for TEM [1]. Among 179 subjects analysed with CBF and beat pattern, 121 subjects had sufficient tissue for epithelial integrity measurements and 159 subjects had tissue processed for ultrastructure analysis. The mean of cross sections examined for each subject was 56 (range: 40–98). The percentages of different cell types observed in the ciliated epithelial strips are summarized in Table 3. There was no significant difference between the percentages of different cells types across age groups (ANOVA, *p* > 0.1). Ciliated cells formed about 50% of the cell population.

**Table 3:** Transmission electron microscopy examination of cell types

Age (years)	N	Ciliated cells (%)	Unciliated cells (%)	Mucous cells (%)	Dead cells (%)
< 18	95	52.0 (0.0, 100.0)	35.8 (0.0, 100.0)	12.2 (0.0, 52.0)	0.0 (0.0, 0.0)
2–6	37	53.6 (0.0, 100.0)	37.1 (0.0, 100.0)	9.3 (0.0, 34.3)	0.0 (0.0, 0.0)
7–12	30	46.0 (0.0, 100.0)	38.3 (0.0, 100.0)	15.7 (0.0, 78.7)	0.0 (0.0, 0.0)
13–17	28	56.4 (0.0, 100.0)	31.3 (0.0, 77.8)	12.3 (0.0, 55.5)	0.0 (0.0, 0.0)
> 18	26	51.0 (17.8, 93.0)	36.5 (3.6, 77.5)	12.3 (0.0, 43.0)	0.2 (0.0, 2.7)

Results are expressed as the mean percentage (5th, 95th percentiles) for each age group.

### Assessment of ciliary epithelial integrity and ultrastructure by transmission electron microscopy

**Fig. 2:**
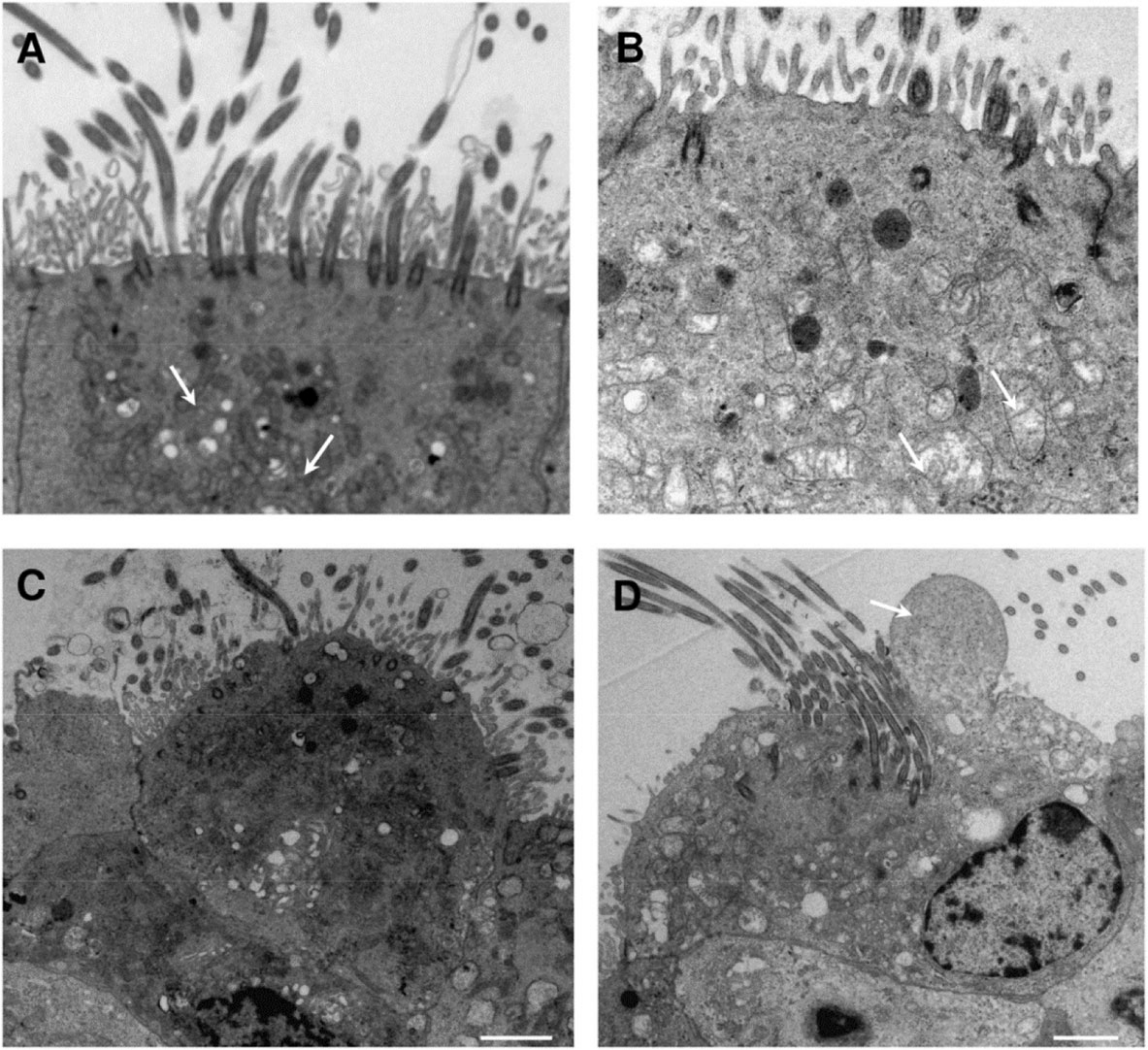
Transmission electron micrographs showing the normal epithelium and the parameters assessed to examine the epithelial damage. **a** Normal with a normal healthy mitochondrion (arrow, bar = 1 μm). **b** Loss of cilia, grade 3, and a cell with a damaged mitochondrion (arrow, bar = 1 μm). **c** Cellular extrusion, grade 2, (bar = 2 μm). **d** Cytoplasmic blebbing, grade 2 (arrow, bar = 2 μm)

**Fig. 3:**
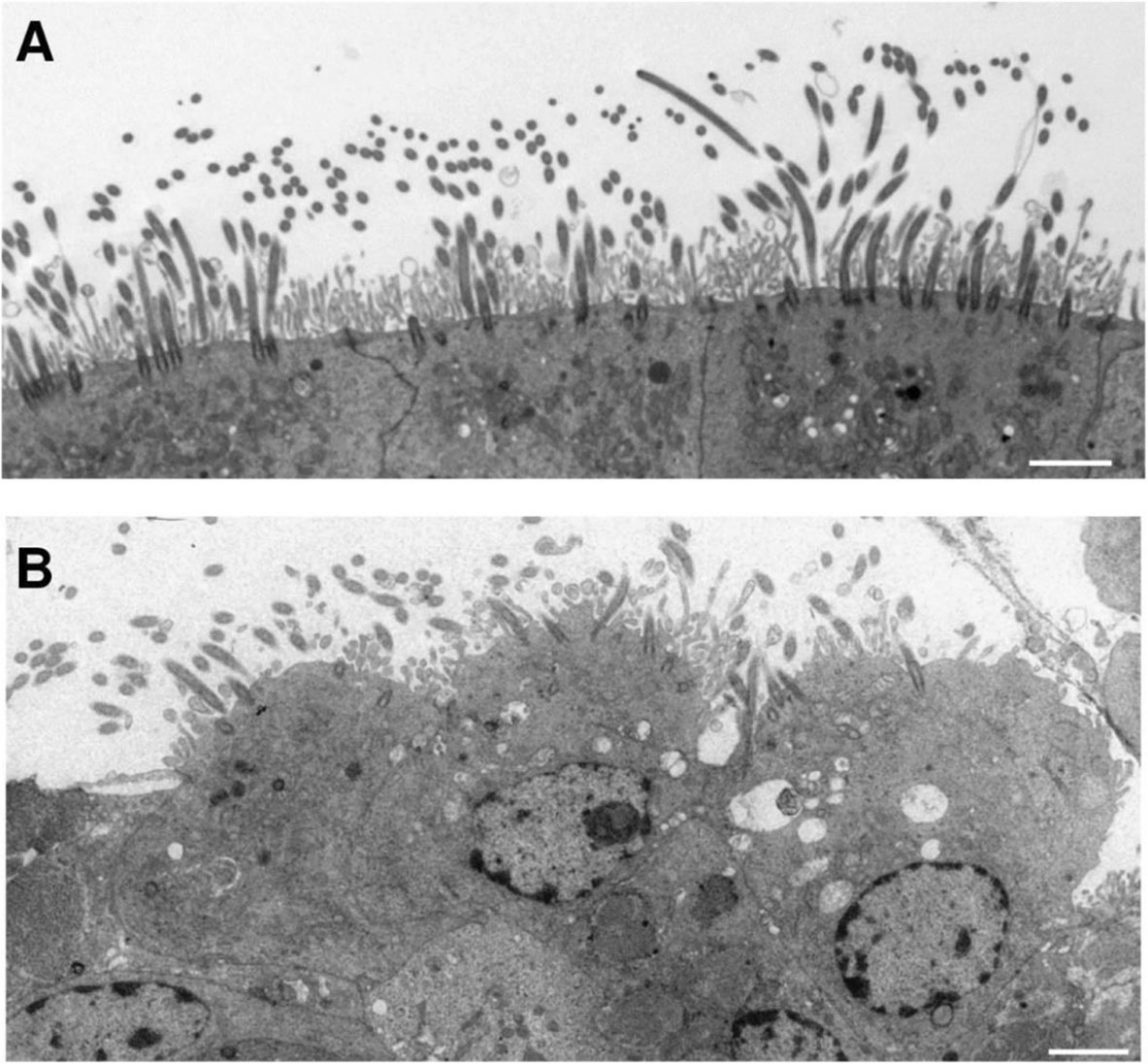
Transmission electron micrographs illustrating the assessment of epithelial integrity. **a** Normal nasal epithelium with an intact ciliated surface and minimal disruption (epithelial integrity score = 0, bar = 2 μm). **b** Abnormal epithelium with severely disrupted cell surface and marked loss of cilia (epithelial integrity score = 4, bar = 2 μm)

The integrity of ciliated epithelium was assessed by examining factors including loss of cilia, cellular extrusion, cytoplasmic blebbing, and mitochondrial damage in Fig. 2. Normal with a normal healthy mitochondrion (arrow, bar = 1 μm) in Fig. 2a. Loss of cilia, grade 3, and a cell with a damaged mitochondrion (arrow, bar = 1 μm) in Fig. 2b. Cellular extrusion, grade 2, (bar = 2 μm) in Fig. 2c. Cytoplasmic blebbing, grade 2 (arrow, bar = 2 μm) in Fig. 2d. Evidence of minor epithelial damage was observed and the analysis is summarised in Table 4. There was no significant difference of epithelial integrity score across age groups (ANOVA, *p* = 0.07). Normal nasal epithelium with an intact ciliated surface and minimal disruption (epithelial integrity score = 0, bar = 2 μm) is shown in Fig. 3a and abnormal epithelium with severely disrupted cell surface and marked loss of cilia (epithelial integrity score = 4, bar = 2 μm) in Fig. 3b.

**Table 4:** Assessment of epithelial integrity by transmission electron microscopy

Age (years)	N	Cells with loss of cilia (%)	Cells extruding from epithelial edge (%)	Cells with cytoplasmic blebbing (%)	Cells with mitochondrial damage (%)	Epithelial integrity
< 18	95	41.4 (0.0, 56.8)	31.5 (0.0, 67.0)	21.1 (0.0, 100.0)	16.3 (0.0, 100.0)	1.83 (0.8, 3.0)
2–6	37	55.1 (0.0, 100.0)	26.1 (0.0, 67.0)	10.8 (0.0, 50.0)	14.9 (0.0, 100.0)	1.8 (0.0, 3.1)
7–12	30	44.4 (0.0, 100.0)	41.0 (0.0, 90.9)	29.2 (0.0, 100.0)	14.4 (0.0, 100.0)	2.1 (0.0, 3.5)
13–17	28	20.3 (0.0, 56.8)	28.6 (0.0, 67.0)	25.9 (0.0, 100.0)	20.2 (0.0, 100.0)	1.6 (0.0, 3.0)
> 18	26	49.3 (0.0, 100.0)	40.2 (6.0, 67.0)	24.7 (0.0, 61.1)	13.4 (0.0, 100.0)	2.0 (1.0, 3.0)

Results are expressed as the mean percentage (5th, 95th percentiles) for each age group.

**Fig. 4:**
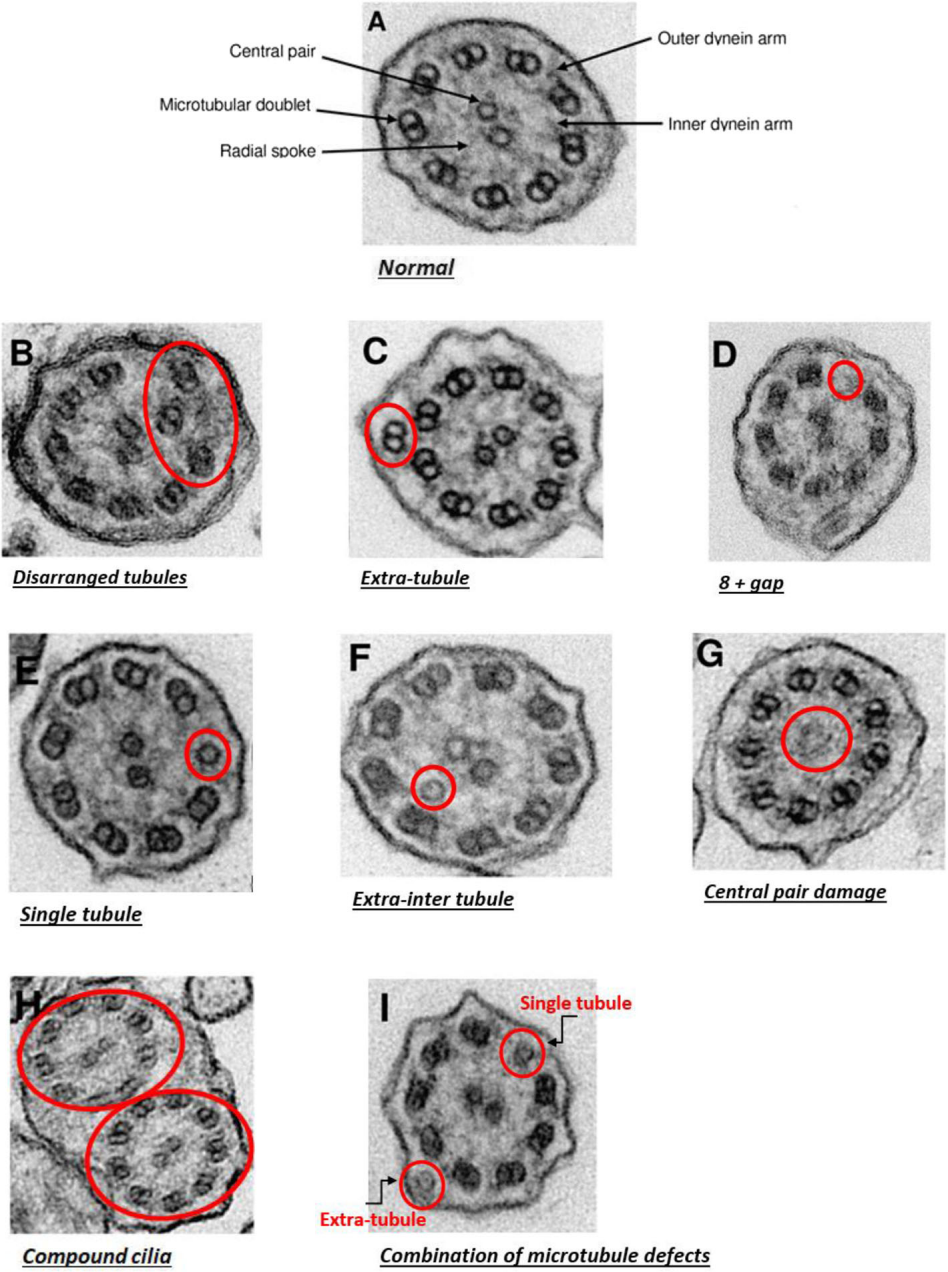
Transmission electron micrographs demonstrating the representative image of ciliary ultrastructural defects. **a** Normal. **b-e** Microtubule defects. **f-g** Central microtubules defects. **h** Compound cilia. **i** Combination of microtubule defects.

Ultrastructural analysis is summarised in Table 5. Abnormal cilia were observed in some subjects. Microtubule defects including disarranged tubules (0.7%), extra-tubule including extra-single tubule and extra-microtubular pair (2.1%), 8 + gap (1.0%) and single tubule (5.5%); central microtubules defects including extra-inter tubule (0.3%) and central pair damage (1.2%); compound cilia (0.5%) and combination of defects (0.9%) were identified in all of the cilia counted in children aged < 18 years (Fig. 4 b-i). The commonest defect identified was single micro-tubule (Fig. 4e).

**Table 5:** Analysis of ciliary ultrastructure by transmission electron microscopy

Age (years)	N	Outers	Inners	Dynein arm defects (%)	Microtubules defects (%)	Central microtubules defects (%)	Compound cilia (%)	Other defects (%)
< 18	121	8.5 (7.5, 9.0)	7.8 (6.5, 8.6)	0.0 (0.0, 0.0)	9.3 (0.0, 28.4)	1.5 (0.0, 9.1)	0.5 (0.0, 5.8)	0.9 (0.0, 6.6)
2–6	46	8.5 (7.8, 9.0)	7.8 (6.9, 8.5)	0.0 (0.0, 0.0)	8.3 (0.0, 27.9)	1.5 (0.0, 10.4)	0.2 (0.0, 0.0)	0.5 (0.0, 7.0)
7–12	36	8.3 (6.9, 9.0)	7.6 (4.2, 8.5)	0.0 (0.0, 0.0)	5.3 (0.0, 22.6)	2.7 (0.0, 19.7)	1.0 (0.0, 9.2)	0.3 (0.0, 3.1)
13–17	39	8.7 (8.3, 9.0)	8.1 (6.7, 8.8)	0.0 (0.0, 0.0)	14.4 (0.0, 37.8)	0.4 (0.0, 3.7)	0.4 (0.0, 6.3)	1.8 (0.0, 11.1)
> 18	38	8.5 (7.0, 9.0)	7.3 (5.8, 8.5)	0.0 (0.0, 0.0)	10.1 (0.0, 31.5)	1.9 (0.0, 16.1)	0.7 (0.0, 6.3)	0.9 (0.0, 13.1)

Results are expressed as the mean percentage (5th, 95th percentiles) for each age group.

The mean microtubule defects in the 13–17 year group (14.4%) were significantly higher than those in the other age groups (8.3% in 2–6 year group, 5.3% in 7–12 year group; ANOVA, *p* < 0.01). Central microtubules defects, compound cilia and other microtubule defects referring to a combination of defects were found in less than 3% of all of the cilia counted in children aged < 18 years (Table 5). There was no significant difference between age groups for the central microtubules defects, compound cilia and other microtubule defects (ANOVA, *p* > 0.05).

## Discussions

We established the reference ranges for ciliary function and structure in healthy Chinese children using HSVM and TEM. We found an overall mean CBF of 10.1 Hz in children aged < 18 years. CBF did not vary with age in these children.

We did not find a significant effect of SHS exposure on CBF of nasal cilia in our children but adults with SHS exposure had a significantly higher mean CBF compared to adults without. Previous studies had been carried out in adults only and the results were conflicting. Nasal CBF in active or passive smokers was significantly lower than that in non-smokers exposed in one study [20]. However, this study included subjects with middle ear diseases and healthy controls. Our findings concurred with more recent studies showing higher mean CBF in nasal epithelial biopsies or air liquid interface (ALI) cultures among active smokers and non-smokers exposed to SHS, compared to non-smokers [21, 22].

We found a lower overall mean CBF in healthy Chinese children compared to the European study [1]. Previous study showed different baseline CBF values between two UK centres that adopted a similar protocol but used different equipment. The investigators believed that the difference in baseline CBF values was genuine but it could not be simply explained by the use of different equipment [16]. Our study adopted exactly the same methodology as one of the two centres. The HK team was trained by the European study team in the UK. A member of the European team visited the laboratory in HK to ensure the methods used were the same, with samples observed using slide chambers and a heated stage which negated differences due to laboratory temperature and humidity. The observed difference could be due to different status of the subjects as the samples were obtained from awake Chinese children, whereas the samples from European children were obtained immediately after anaesthetic induction for elective surgery. However, Propofol used in the anaesthetic induction was not shown to affect CBF [23]. CBF increased rapidly during the first 3 h and reduced after 9 h from the time of sampling. Most of our samples were processed within the plateau phase of measurement and there was no significant effect of processing time on the mean CBF among our subjects [19]. Subclinical laboratory-confirmed respiratory infections due to respiratory syncytial virus (RSV) and influenza are common in children during influenza seasons in HK [24, 25] and they might have affected ciliary function in some cases. Yet, similar exclusion criteria to the European study were used to minimise this effect. The other possibility was an observer bias but the difference of CBF of our intra-observer and inter-observer mean was smaller than the European study. Our findings cannot negate the possibility of a genuine genetically determined difference between European and Chinese children. Further study may be warranted for elucidation. However, the wide ranges of maximum and minimum CBF, the higher CBF in smokers and the difference between our centre and the European study using exactly the same methodology support that CBF alone should not be used for diagnosing PCD.

One patient had a mixed ciliary beat pattern with dyskinetic cilia, cilia with normal pattern and cilia with circular beat pattern on occasional ciliated cells. Circular beat pattern is observed in ciliary trans-position defect and central microtubular agenesis [8, 26], but the cilia of all the ciliated cells show dyskinetic beat pattern when viewed from above in these cases. We invited the subject to repeat the test, which showed normal CBF and normal beat pattern in 3 different beating planes including side-view profile, towards the observer and view from above [27]. The percentage of dyskinetic edge decreased from 33.3 to 10%. Thus, we believed that the initial abnormal finding was secondary in nature, possibly due to a preceding subclinical respiratory infection.

Our results of ultrastructural analysis were in agreement with other reports on the number of outer and inner dynein arms visible, number of ciliated cells and epithelial integrity [1, 28]. Nevertheless, we found a higher percentage of microtubular defects, especially in the 13–17 year age group compared to the European study. The reasons for this are unclear. Some children might have had unrecognized subclinical mild respiratory infection or might have been in the recovery phase. Microtubular defects were reported in the recovery phase of mild respiratory infection in some patients up to 10 weeks after acute phase [29, 30]. Damage to the nasal ciliated epithelium might also occur during sampling. Smoking and other tobacco use may have an effect on ciliary function [31, 32]. We may have included adolescents that smoked or used other tobacco products, although it is not likely as the local prevalence of recent cigarette smoking and other tobacco use in 45,857 secondary school students is low, with 3.3% reported cigarettes use, 1.1% reported e-cigarette use and 3% reported other tobacco use in the past 30 days [33]. Culture of ciliated epithelial cells at ALI, with re-differentiation and re-analysis of ciliary function and ultrastructure may eliminate secondary damage but was not feasible during this study [34]. As such, ERS task force recommends to proceed with ALI culture or obtain 3 separate biopsies on separate visits if PCD is highly suspected [4, 16]. Due to resource limitations, we could only invite the subject with circular beat pattern to repeat the test to ensure that we had not included a PCD case in the normal reference study.

We had a large sample size of healthy subjects and none were on medication. Our methodology was robust as we followed the European study [1] and were closely supervised. Our study was conducted to avoid peak seasons for viral infections. However, laboratory surveillance data showed influenza was still prevalent in March 2016 with adenovirus, RSV and rhinovirus circulating in our community for that whole year [35]. We might have included some subjects with subclinical infections or recovering from very mild infection. We also identified subjects with undiagnosed AR and asthma. We excluded them in the analysis but might still have included some individuals with very mild AR and local inflammation leading to secondary ciliary dyskinesia.

## Conclusion

In conclusion, we established normal reference ranges for respiratory ciliary function and ultrastructure in healthy Chinese children. We found a lower mean CBF in our children aged < 18 years compared to a European study that used the same methodology. The reasons remain uncertain but suggest the necessity to establish normal reference ranges for different centres. In addition, the wide ranges of maximum and minimum CBF and higher CBF in smokers despite our rigorous methodology also support that CBF alone should not be used for diagnosing PCD.

## Data Availability

The datasets used and/or analysed during the current study are available from the corresponding author on reasonable request.

## Abbreviations

PCD: Primary ciliary dyskinesia
ERS: European Respiratory Society
nNO: Nasal nitric oxide
HSVM: High speed video microscopy
CBF: Ciliary beat frequency
ATS: American Thoracic Society
TEM: Transmission electron microscopy
UK: United Kingdom
HK: Hong Kong
URTI: upper respiratory tract infection
SHS: Second hand smoking
SD: Standard deviation
ANOVA: Analysis of variance
CI: Confidence interval
AR: Allergic rhinitis
ALI: Air liquid interface
RSV: Respiratory syncytial virus
HKU/HA HKWC: The University of Hong Kong/Hospital Authority Hong Kong West Cluster
